# Healthcare-Associated Conjunctivitis in the NICU: Microbiological Spectrum, Antimicrobial Resistance and Treatment Patterns

**DOI:** 10.3390/pathogens15020209

**Published:** 2026-02-13

**Authors:** Hatice Turgut, Elif Seren Tanrıverdi, Eda Karadoğan, Ramazan Özdemir

**Affiliations:** 1 Department of Pediatrics, Division of Neonatology, Faculty of Medicine, Inonu University, 44280 Malatya, Türkiye; 2 Department of Medical Microbiology, Faculty of Medicine, Inonu University, 44000 Malatya, Türkiye; 3 Division of Epidemiology, Antalya Provincial Health Administration, Republic of Turkey Ministry of Health, 07040 Antalya, Türkiye

**Keywords:** healthcare-associated conjunctivitis, neonates, fortified eye drops, Gram-positive bacteria, antimicrobial resistance

## Abstract

Healthcare-associated bacterial conjunctivitis is an underrecognized yet preventable infection in neonatal intensive care units (NICUs). This study aimed to determine the incidence, risk factors, microbiological profile, and treatment approaches of healthcare-associated bacterial conjunctivitis in neonates. This descriptive, cross-sectional study included neonates diagnosed with bacterial conjunctivitis and followed in the NICU between January 2019 and January 2024. Pathogens were identified by MALDI-TOF MS and antimicrobial susceptibility determined using VITEK 2 according to EUCAST breakpoints. During the five-year period, 104 (2.5%) of 4107 neonates admitted to the NICU developed healthcare-associated bacterial conjunctivitis. Of the pathogens isolated in cultures, 70.2% were Gram-positive bacteria, with coagulase-negative staphylococci being the most common (52.9%), followed by *Staphylococcus aureus*, *Klebsiella pneumoniae*, and *Serratia marcescens*. Empirical treatment consisted of 0.3% topical gentamicin eye drops. In resistant cases, fortified vancomycin drops (32.7%), 0.5% moxifloxacin (4.8%), or 0.3% tobramycin (1.9%) eye drops were administered according to antibiogram results. Compared with Gram-positive infections, Gram-negative conjunctivitis was associated with longer durations of intubation, orogastric feeding, and hospitalization. These findings indicate a predominance of Gram-positive pathogens in NICU-acquired neonatal conjunctivitis, while Gram-negative infections confer greater clinical burden. Fortified antibiotic eye drops are an effective treatment option for resistant cases in high-risk newborns.

## 1. Introduction

Bacterial conjunctivitis, characterized by inflammation and bacterial proliferation of the conjunctival epithelium, is the most common ocular infection. In neonatal intensive care units (NICUs), bacterial conjunctivitis represents a significant source of healthcare-associated infections and ocular morbidity among vulnerable newborns [1,2].

The incidence of conjunctivitis in neonates has increased due to several predisposing factors, including underdeveloped lacrimal ducts and eyelid function, low tear secretion, an immature immune system, frequent therapeutic interventions that may cause mechanical trauma, contamination of the conjunctival mucosa with infected secretions in patients receiving respiratory support, and prolonged hospitalization [2,3,4]. Surveillance studies have reported the incidence of healthcare-associated conjunctivitis to range from 5% to 14.5%, underscoring its clinical significance in NICUs [5].

The prognosis of healthcare-associated conjunctivitis in NICUs is generally favorable. However, depending on the causative microorganism, the infection may lead to ocular complications, as well as systemic complications such as sepsis, pneumonia, and meningitis [3]. Moreover, it poses a risk for horizontal transmission within the NICU. Therefore, early diagnosis and appropriate treatment are essential [5,6]. Conjunctivitis is typically managed effectively within 7–10 days using empirical topical antibiotic therapy. Such treatment minimizes disease severity, transmission risk, complications, and the likelihood of reinfection [2,7]. Nevertheless, the rising antimicrobial resistance among neonatal pathogens may render empirical therapy ineffective, emphasizing the importance of culture-based, susceptibility-guided treatment adjustments.

Despite the ubiquity of empiric topical regimens in NICUs, rising and unit-specific resistance among common neonatal ocular pathogens undermines “one-size-fits-all” therapy. Evidence specific to neonates on the safety, dosing, and effectiveness of fortified topical antibiotics remains limited and is often extrapolated from older pediatric or adult keratitis/endophthalmitis series rather than culture-confirmed neonatal cohorts. As a result, culture-based, susceptibility-guided adjustments are not consistently deployed early, which may prolong symptoms and supportive care needs.

To address this gap, we analyzed a five-year, culture-confirmed NICU cohort to define incidence, contemporary microbiology, and resistance patterns; compared clinical burden by Gram-stain category; and described real-world escalation from empiric gentamicin to fortified, culture-directed therapy. Our aim is to deliver pragmatic, unit-level evidence that optimizes initial empiric choices and supports timely transition to targeted treatment in high-risk neonates.

## 2. Materials and Methods

### 2.1. Study Design and Patient Population

This retrospective descriptive cohort study included neonates who were admitted to the NICU between January 2019 and January 2024 and diagnosed with healthcare-associated bacterial conjunctivitis. The diagnosis was established according to the criteria defined by the Centers for Disease Control and Prevention (CDC) [8].

### 2.2. Inclusion and Exclusion Criteria

Neonates who met the CDC diagnostic criteria for conjunctivitis and had a positive result in conjunctival swab culture were included in the study. Cases in which multiple microorganisms were isolated and considered contamination, patients whose conjunctival swab samples were collected within the first 48 h of hospitalization, and those whose clinical findings resolved before the initiation of antibiotic therapy were excluded from the study.

### 2.3. Sampling and Data Collection

No sampling method was applied; all patients meeting the inclusion criteria were enrolled in the study. Patient data were retrospectively retrieved from electronic medical records. Demographic variables (gestational age, birth weight, mode of delivery, sex), maternal diseases, presence of premature rupture of membranes, reason for NICU admission, need for resuscitation at birth, duration of intubation, nasal continuous positive airway pressure (NCPAP) therapy, and duration of orogastric tube feeding were recorded. Additionally, whether the infant had undergone examination for retinopathy of prematurity (ROP) prior to the onset of conjunctivitis and whether phototherapy or antibiotic treatment had been administered were noted. The onset time of ocular discharge was documented. Microbiological findings were categorized according to Gram-staining characteristics, and antibiotic susceptibility results were recorded. The therapeutic agent used and duration of treatment were documented in the data collection form.

According to institutional protocol, neonates who met the CDC diagnostic criteria and did not receive systemic antibiotic therapy were empirically treated with 0.3% gentamicin eye drops (one drop, four times daily). In cases of Gram-negative infection resistant to gentamicin based on antibiotic susceptibility results, treatment was modified accordingly. For Gram-positive cases presenting with aminoglycoside and second- or third-generation fluoroquinolone resistance, persistent purulent discharge, poor clinical response, or strong clinical suspicion of conjunctivitis, fortified topical vancomycin drops (25 mg/mL) were initiated. The fortified vancomycin solution was prepared under aseptic conditions, stored at +4 °C, renewed every five days, and administered using sterile single-use syringes (one drop, four times daily) [9]. The duration of treatment varied between 7 and 10 days, depending on clinical response.

In infants with suspected sepsis, blood, cerebrospinal fluid and urine cultures were obtained as part of the routine sepsis evaluation according to unit protocols. Culture-confirmed sepsis was defined as the presence of a positive culture in at least one of these samples (blood, cerebrospinal fluid or urine). Clinical sepsis was defined by compatible clinical and laboratory findings in the absence of culture positivity.

### 2.4. Study Setting

The study was conducted in a tertiary care hospital with a capacity of 1600 beds, including a 58-bed NICU. The NICU provides tertiary-level care for approximately 900 neonates annually and also accepts referrals from neighboring provinces.

### 2.5. Microbiological Analysis

In our hospital, sterile conjunctival swab samples are collected from neonates in the NICU with suspected conjunctivitis. After cleansing the periorbital area with sterile gauze and normal saline, the culture specimen is obtained from the lower conjunctival sac using a sterile cotton swab (microcult^®^ Transport Swabs, Beijing, China).

To prevent contamination, contact with surrounding tissues is strictly avoided. The collected samples are transported to the microbiology laboratory in appropriate transport media, where Gram staining, culture, and antibiotic susceptibility testing are performed.

Bacterial identification was performed using the MALDI-TOF MS system (bioMérieux, Marcy-l’Etoile, France), and antimicrobial susceptibility testing was carried out with the VITEK 2 automated system (bioMérieux, Marcy-l’Etoile, France). Results were interpreted according to the clinical breakpoint values established by the European Committee on Antimicrobial Susceptibility Testing (EUCAST). Methicillin resistance was assessed only for *Staphylococcus* spp. isolates, while *Enterococcus faecalis* isolates were excluded from this analysis.

Quantitative microbial counts were not routinely performed for conjunctival swab cultures in our laboratory. Therefore, in isolates identified as coagulase-negative staphylococci (CoNS), differentiation between true infection and conjunctival colonization was based on a combination of clinical findings (purulent discharge, conjunctival hyperemia, and eyelid edema) and microbiological criteria, including consistent growth of the same CoNS species in repeat conjunctival cultures, identical MALDI-TOF MS identification, and concordant antimicrobial susceptibility profiles. Isolates fulfilling these criteria were considered clinically significant and included in the final analysis, whereas those not meeting the criteria were classified as colonization and excluded from the analysis.

### 2.6. Statistical Analysis

Descriptive statistics are presented as frequencies and percentages for categorical variables and as mean ± standard deviation or median (min–max) for continuous variables, depending on the data distribution. The distribution of continuous variables was assessed using the Kolmogorov–Smirnov test, Shapiro–Wilk test, coefficient of variation, detrended normal Q-Q plots, and histograms. Categorical variables were compared using the chi-square test or Fisher’s exact test. Continuous variables between two groups were compared using Student’s *t*-test when the assumption of normality was met, and the Mann–Whitney *U* test when this assumption was not satisfied in at least one group.

Missing data were excluded from analysis without imputation, and all analyses were performed on complete cases only. A *p*-value < 0.05 was considered statistically significant. Effect sizes are expressed as odds ratios (ORs) with 95% confidence intervals. All statistical analyses were performed using IBM SPSS Statistics for Windows, version 25.0 (IBM Corp., Armonk, NY, USA), accessed in January 2026.

Variables included in the binary logistic regression model were selected a priori based on clinical relevance and previous literature, as well as variables showing an association with Gram-negative etiology in univariate analyses. To reduce model complexity and minimize the risk of overfitting given the sample size, the number of covariates included in the model was deliberately limited.

## 3. Results

### 3.1. Patient Characteristics

During the five-year study period, 104 of 4107 neonates (2.5%) admitted to the NICU met the inclusion criteria and were included in the study. Of these patients, 57.7% were male, 74% were born before 36 weeks of gestation, and 74% had a birth weight below 2500 g. The majority of infants (91.7%) were delivered by cesarean section. More than half of the patients (55.8%) were admitted to the NICU due to prematurity, followed by respiratory distress (9.6%) and congenital anomalies (11.5%) as the next most common reasons for admission (Table 1).

Prior to the onset of conjunctivitis, 57.4% of the patients (27/47) had received phototherapy, and 73.3% (11/15) had undergone ophthalmologic examination for ROP. During hospitalization, recurrent conjunctivitis developed in 9.6% (n = 10) of the patients, while 16.3% (n = 17) had concurrent clinically or culture-confirmed sepsis. Seventeen infants who developed concomitant sepsis constituted a high-risk subgroup. Of these infants, 64.7% were born before 36 weeks of gestation and 70.6% had a birth weight below 2500 g. Premature rupture of membranes was present in 70.6% of cases, and most deliveries were by cesarean section (94.1%).

Among these patients, culture-confirmed sepsis was documented in 10 infants based on positive blood culture results, whereas the remaining 7 infants were classified as having clinical sepsis.

The onset of conjunctivitis symptoms and signs occurred between days 3–7 in 26 patients, during weeks 2–3 in 34 patients, weeks 3–4 in 19 patients, weeks 4–5 in 12 patients, and after the 5th week in 13 patients.

### 3.2. Microbiological Findings

**Figure 1:**
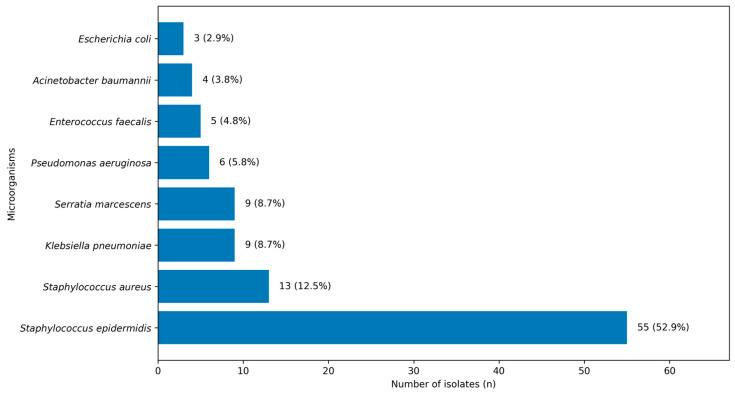
Distribution of microorganisms isolated from conjunctival swab cultures.

Gram-positive organisms accounted for 70.2% (n = 73) of the isolates, while Gram-negative organisms comprised 29.8% (n = 31). The most frequently identified pathogen was *Staphylococcus epidermidis* (52.9%), followed by *Staphylococcus aureus* (12.5%), *Klebsiella pneumoniae* (8.7%), and *Serratia marcescens* (8.7%) (Figure 1). Only *S. epidermidis* isolates that demonstrated repeat growth in subsequent cultures and were deemed clinically significant were considered true pathogens.

Among the 17 infants with concomitant sepsis, culture-confirmed sepsis was identified in 58.8% (n = 10) of cases. The most frequently isolated organisms from systemic cultures were *Acinetobacter baumannii* (40%) and *Pseudomonas aeruginosa* (20%), followed by *Staphylococcus epidermidis* (20%), *Klebsiella pneumoniae* (10%), and *Serratia marcescens* (10%).

### 3.3. Antimicrobial Susceptibility

Overall, 51.9% of the isolates were susceptible to gentamicin, while 48.1% exhibited resistance. Among the resistant microorganisms, 82.0% were Gram-positive. The methicillin susceptibility rate among *Staphylococcus* spp. isolates was 57.4% (Table 2A). Most Gram-negative isolates were susceptible to carbapenems; however, a limited number demonstrated carbapenem resistance (Table 2B). Carbapenem resistance was found to be significantly higher among non-*Enterobacterales* isolates compared with *Enterobacterales* species (*p* < 0.05). Antibiotic susceptibility patterns for Gram-positive and Gram-negative pathogens are summarized in Table 2A,B.

### 3.4. Treatment

All patients initially received empirical topical 0.3% gentamicin therapy. In cases resistant to gentamicin (n = 50; 48.1%), treatment was adjusted according to antibiotic susceptibility results. Fortified vancomycin eye drops were administered to 32.7% of patients (n = 34), 0.5% moxifloxacin to 4.8% (n = 5), and 0.3% tobramycin to 1.9% (n = 2). In 16.3% of cases (n = 17), treatment was continued with a systemic antibiotic to which the causative organism was susceptible.

All systemic antibiotics administered (amikacin, meropenem, colistin, and vancomycin) were selected based on antimicrobial susceptibility testing and were active against both systemic and conjunctival pathogens. Given the presence of prematurity, low birth weight, and concurrent sepsis with potential multidrug-resistant organisms, conjunctivitis was considered part of a systemic infection rather than an isolated local condition; therefore, treatment was continued with pathogen-directed systemic antibiotics without additional topical therapy.

### 3.5. Clinical Outcomes

Compared with patients who had Gram-positive conjunctivitis, those with Gram-negative infections exhibited significantly longer durations of intubation, orogastric tube feeding, and hospitalization (*p* < 0.001, *p* = 0.011, and *p* = 0.023, respectively; Table 3). To identify factors independently associated with Gram-negative etiology, a binary logistic regression analysis was subsequently performed (Table 4).

## 4. Discussion

Healthcare-associated conjunctivitis, defined as conjunctivitis developing ≥48 h after hospital admission, is a preventable healthcare-associated infection that contributes to ocular morbidity in NICUs. The reported prevalence of healthcare-associated conjunctivitis ranges between 1.6% and 12% [2]. In the present study, the incidence of healthcare-associated conjunctivitis was found to be 2.5%. The absence of specific diagnostic guidelines for neonatal conjunctivitis and the subtle presentation of symptoms other than discharge—such as pain, redness, or swelling—particularly in premature infants, make diagnosis challenging and contribute to variability in incidence rates [3,10]. Furthermore, frequent conjunctival colonization among NICU patients and the difficulty in distinguishing colonization from true infection in this population also account for differences in reported incidence [2,11].

Prematurity and low birth weight are well-established risk factors for neonatal infections. In premature infants, an underdeveloped lacrimal system, immature immunity, exposure to invasive procedures, mechanical trauma during therapeutic interventions, prolonged hospitalization, and antibiotic use further increase this risk. In our cohort, 70% of infants had low birth weight and were born before 36 weeks’ gestation, and 55.8% were admitted due to prematurity. Comparable studies have reported that the proportion of healthcare-associated conjunctivitis among preterm infants is 80% and 75.4%, respectively [3,10,12].

In 11.6% of patients, conjunctivitis developed following ROP examination, and in 26% it occurred after phototherapy. As the eyelids open and close, they function like a pump, facilitating the drainage of epithelial debris and bacteria together with tears. During phototherapy, ocular occlusion keeps the eyelids closed, thereby increasing the risk of conjunctivitis and bacterial colonization in the conjunctival sac [12,13]. In our cohort, Gram-positive pathogens were more frequently identified among cases developing conjunctivitis after ROP assessment or phototherapy; however, these associations did not reach statistical significance (*p* = 0.167 and *p* = 0.642, respectively).

When the relationship between respiratory and nutritional support and conjunctivitis was examined, 73% of patients received nasal CPAP support, 30.8% required mechanical ventilation, and 93.3% were fed via an orogastric catheter. Comparing the durations of these supports by Gram-stain category, the durations of orogastric catheter use and endotracheal intubation were longer in Gram-negative infections. In this vulnerable patient population, conjunctival infection is more common due to contamination of the conjunctiva with gastrointestinal and respiratory secretions, as well as the increased frequency of interventions and manipulations by caregivers [10,12].

In our cohort, 70.2% of pathogens were Gram-positive and 29.8% were Gram-negative bacteria. In comparison, Pak et al. reported a Gram-positive conjunctivitis rate of 81.7% (67/82), and Tang XJ et al. reported 71.1% (54/76) [4,14,15]. In a 14-year time-trend analysis by Tang S. et al., Gram-positive organisms accounted for 74.5% (298/400) of all cases; although the overall proportion of Gram-positive conjunctivitis declined from 82.1% to 74.5% over time, the share attributable to CoNS increased [15]. Consistent with these studies, CoNS was the most frequently isolated pathogen in our series and was considered clinically significant based on concordant clinical signs of conjunctivitis and repeat positive conjunctival cultures. While CoNSs are commonly regarded as part of the normal conjunctival microbiota—frequently detected on the skin and within the conjunctival sac and often nonpathogenic—in neonates, they may acquire pathogenicity. Immature immune defenses, inadequate mucosal barriers, increased antibiotic exposure, and colonization of medical devices (e.g., nCPAP cannulae, endotracheal tubes, and orogastric catheters) with subsequent transfer to the conjunctiva can facilitate progression from colonization to overt infection. In the present study, CoNS isolates were classified as true pathogens only when supported by compatible clinical findings and confirmed by repeat conjunctival cultures yielding identical MALDI-TOF MS identification and concordant antimicrobial susceptibility profiles. This conservative approach was deliberately adopted to minimize misclassification and to reduce the risk of overestimating the contribution of colonizing flora to healthcare-associated conjunctivitis. Accordingly, the predominance of CoNS in our cohort should be interpreted in the context of strict microbiological and clinical criteria rather than simple isolation from conjunctival swabs.

Purulent conjunctival discharge may serve as a clinical clue for late-onset sepsis, particularly in low-birth-weight neonates. Accordingly, infants with purulent discharge should be closely monitored for sepsis during periods of respiratory and nutritional support. In our study, late-onset sepsis associated with conjunctivitis was observed in 16.3%, and Gram-negative conjunctivitis was significantly more frequent in these cases (*p* < 0.001). Because the systemic antibiotics initiated in these infants were active against the isolated pathogens, no additional topical therapy was administered. In a study evaluating late-onset sepsis in the presence of conjunctival discharge, 6.2% of cases showed a relationship between positive conjunctival cultures and sepsis (*p* = 0.543), and 70% of the identified pathogens were Gram-negative bacteria [5].

Previous studies have demonstrated that coagulase-negative staphylococci (CoNSs) exhibit a progressively declining susceptibility to commonly used topical antibiotics, while retaining susceptibility to vancomycin [1,11]. Our findings are consistent with this pattern, showing that CoNS isolates display limited responsiveness to widely prescribed topical agents such as aminoglycosides and fluoroquinolones, whereas vancomycin remains consistently effective. These concordant results further underscore the reliability of vancomycin in CoNS infections and draw attention to the growing significance of antimicrobial resistance in the selection of empirical and topical treatment strategies. In cases of resistant CoNS growth (n = 34; 32.7%) and persistent clinical signs despite treatment, fortified vancomycin eye drops were initiated; no local adverse reactions were observed, and the clinical findings resolved with therapy. Fortified topical preparations are preferred treatment for keratitis and endophthalmitis and, particularly for multidrug-resistant strains, may serve as an alternative to systemic therapy in high-risk groups such as neonates and pediatric patients [9].

Our study has several limitations. As a single-center clinical study, nevertheless, we believe that the results are valuable for tertiary care institutions with patient profiles similar to ours. In addition, because the data were obtained retrospectively from electronic medical records, some antimicrobial susceptibility tests could not be performed for all isolates. Furthermore, the relatively small number of Gram-negative cases limited the complexity of multivariable modeling and may increase the risk of overfitting. Therefore, the results of the logistic regression analysis should be interpreted cautiously and primarily as hypothesis-generating rather than definitive evidence of causality.

The strength and originality of our study derive from its comprehensive evaluation of healthcare-associated bacterial conjunctivitis over a five-year period in a tertiary-level neonatal intensive care unit. Unlike previous studies, this research systematically analyzed not only the incidence, microbiological distribution, and antimicrobial resistance patterns but also the clinical predictive factors. The integration of microbiological data with clinical parameters provides a unique contribution to the literature on risk stratification and optimal therapeutic strategies for conjunctivitis in high-risk neonatal populations.

## 5. Conclusions

In conclusion, our findings demonstrate a clear epidemiological shift in healthcare-associated neonatal bacterial conjunctivitis from Gram-negative to Gram-positive pathogens. However, in the presence of premature rupture of membranes and concurrent systemic infection, empirical treatment should prioritize coverage for Gram-negative organisms. The data obtained contribute to the limited international literature on neonatal conjunctivitis in intensive care settings and provide practical guidance for refining empirical treatment algorithms and infection control strategies in similar healthcare environments. Fortified topical antibiotics can be considered a safe and effective option in cases of multidrug-resistant or treatment-refractory infections. Early diagnosis, regular microbiological surveillance, and rigorous antibiotic stewardship are crucial for preventing complications and minimizing horizontal transmission within neonatal intensive care units.

## Figures and Tables

**Table 1:** Demographic and Clinical Characteristics of Neonates with Healthcare-Associated Conjunctivitis.

Variables	n = 104
Sex *	
Female	44 (42.3)
Male	60 (57.7)
Gestational age *	
<36 weeks	77 (74.0)
≥37 weeks	27 (26.0)
Birth weight *	
<2500 g	77 (74.0)
≥2500 g	27 (26.0)
Percentile *	
AGA	75 (72.1)
SGA	24 (23.1)
LGA	5 (4.8)
Mode of delivery *	
Cesarean section	95 (91.3)
Vaginal delivery	9 (8.7)
Premature rupture of membranes *	17 (16.3)
Indication for hospitalization *	
Prematurity	58 (55.8)
Respiratory distress	10 (9.6)
Congenital anomaly	12 (11.5)
Chromosomal anomaly	7 (6.7)
Asphyxia	5 (4.8)
Other	12 (11.5)
History of phototherapy *^a^	27 (57.4)
History of ROP examination *^b^	11 (73.3)
Birth weight (g) **	1982 (500–5200)
Gestational age (weeks) **	34 (25–40)
Ventilator days **	0 (0–40)
NCPAP days **	4 (0–70)
OG feeding days **	15 (0–20)
Length of hospital stay (days) **	30 (5–142)

* n (%) ** median (min–max). ^a^ Evaluated among 47 neonates with available data. ^b^ Evaluated among 15 neonates with available data. Abbreviations: AGA, appropriate for gestational age; SGA, small for gestational age; LGA, large for gestational age; ROP, retinopathy of prematurity; NCPAP, nasal continuous positive airway pressure; C/S, cesarean section; NSVD, normal spontaneous vaginal delivery; OG, orogastric.

**Table 2:** (**A**) Antimicrobial susceptibility of Gram-positive isolates causing healthcare-associated neonatal conjunctivitis (n = 73). (**B**) Antimicrobial susceptibility of Gram-negative isolates causing healthcare-associated neonatal conjunctivitis (n = 31).

(A)			
Antibiotic	*Staphylococcus* spp. (n = 68)	*Enterococcus* spp. (n = 5)	Total (n = 73)
Gentamicin *	30 (44.1)	2 (40)	32 (43)
Erythromycin	20 (29.4)	1 (20)	21 (28)
Tobramycin	27 (39.7)	1 (20)	28 (38)
Amikacin	51 (75)	3 (60)	54 (75)
Trimethoprim–sulfamethoxazole	39 (57.4)	5 (100)	44 (59)
Ciprofloxacin	24 (35.3)	3 (60)	27 (36)
Methicillin	39 (57.4)	N/A	39 (53)
Vancomycin	68 (100)	5 (100)	73 (100)
(**B**)			
**Antibiotic**	***Enterobacterales* (n = 21)**	**Non-*Enterobacterales* (n = 10)**	**Total (n = 31)**
Gentamicin	21 (95.5)	21 (95.5)	22 (70)
Tobramycin	14 (70)	6 (30)	20 (80)
Amikacin	20 (76.9)	6 (23.1)	26 (83)
Trimethoprim–sulfamethoxazole	16 (94.1)	1 (5.9)	17 (62)
Ciprofloxacin	17 (100)	0 (0)	17 (70)
Meropenem	19 (76)	6 (24)	25 (80)
Imipenem	19 (95.0)	1 (5.0)	20 (65)

Data are presented as n (% of isolates within each column). * For *Enterococcus* spp., gentamicin susceptibility indicates the absence of high-level aminoglycoside resistance. Percentages are calculated within each column. Only antibiotics with established clinical relevance for Gram-negative organisms are shown.

**Table 3:** Distribution of Etiologic Agents by Clinical Characteristics in Neonates with Healthcare-Associated Conjunctivitis.

Clinical Characteristic	Gram-Positive Isolates	Gram-Negative Isolates	OR (95% CI)	*p*
Female, n (%)	31 (42.5)	13 (41.4)	1.0 (0.4–2.4)	0.960
Birth weight (g), Median (IQR)	2000 (1188)	1965 (1400)	-	0.691
Gestational age (weeks), Mean ± SD	34 ± 3.7	33 ± 4.1		0.396
<36 weeks, n (%)	55 (75.3)	22 (71)	1.3 (0.5–3.2)	0.642
Cesarean delivery, n (%)	66 (90.4)	29 (93.5)	0.7 (0.1–3.3)	0.721
Premature rupture of membranes, n (%)	7 (9.6)	10 (32.3)	4.5 (1.5–13.3)	0.004
ROP examination, n (%)	10 (13.7)	1 (3.2)	0.2 (0.1–1.7)	0.167
Phototherapy, n (%)	18 (24.7)	9 (29.0)	1.3 (0.5–3.2)	0.642
Concomitant systemic infection, n (%)	5 (6.8)	12 (38.7)	8.6 (2.7–27.4)	<0.001
Duration of intubation (days), Median (IQR)	0 (0)	1 (10)	-	<0.001
NCPAP duration (days), Median (IQR)	3 (10)	6 (26)	-	0.238
OG feeding duration (days), Median (IQR)	10 (26)	28 (27)	-	0.011
Onset of conjunctivitis (days), Median (IQR)	14 (14)	14 (16)	-	0.677
Length of hospital stay (days), Median (IQR)	24 (27)	36 (24)	-	0.023

Abbreviations: OR, odds ratio; CI, confidence interval; SD, standard deviation; IQR, interquartile range. Column percentages are shown.

**Table 4:** Factors Associated with Gram-Negative Etiology in Healthcare-Associated Neonatal Conjunctivitis: Binary Logistic Regression.

Variable	OR (95% CI)	*p*
Premature rupture of membranes	4.1 (1.3–13.0)	0.020
Concomitant systemic infection	8.0 (2.4–26.5)	<0.001
Vaginal delivery (Reference = C/S)	0.8 (0.1–4.4)	0.764

Hosmer–Lemeshow test: Sig. = 0.941; Nagelkerke R^2^ = 0.253. Abbreviations: OR, odds ratio; CI, confidence interval; C/S, cesarean section.

## Data Availability

The data supporting the findings of this study are available from the corresponding author upon reasonable request.

## Abbreviations

The following abbreviations are used in this manuscript:

AGA	Appropriate for gestational age
CDC	Centers for Disease Control and Prevention
C/S	Cesarean section
CoNS	Coagulase-negative staphylococci
LGA	Large for gestational age
NICU	Neonatal intensive care unit (plural: NICUs)
NCPAP	Nasal continuous positive airway pressure
NSVD	Normal spontaneous vaginal delivery
OG	Orogastric
ROP	Retinopathy of prematurity
SGA	Small for gestational age

## References

[1] Suhas P., Vishnu S., Muthayya M. (2021). Pathogenic bacteria and their antibiotic sensitivity in ophthalmia neonatorum. Oman J. Ophthalmol..

[2] Haas J., Larson E., Ross B., See B., Saiman L. (2005). Epidemiology and diagnosis of hospital-acquired conjunctivitis among neonatal intensive care unit patients. Pediatr. Infect. Dis. J..

[3] Goel K., Randhawa V.S., Sail A., Khare S., Kumar A., Dutta R., Goel G. (2016). Incidence, etiology and risk factors associated with neonatal healthcare-associated conjunctivitis: A prospective study from a tertiary care hospital in India. J. Trop. Pediatr..

[4] Pak K.Y., Kim S.I., Lee J.S. (2017). Neonatal bacterial conjunctivitis in Korea in the 21st century. Cornea.

[5] Gad A., Khalil A., Halil M., Chandra P., Soliman A., Rahman E’mar A., Ibrahim M., Al Khzzam F., AlHendawi T., Hamed M. (2023). Preterm infants with positive conjunctival swab culture: Risk factors and association with late-onset sepsis—A retrospective cohort study. Front. Pediatr..

[6] Chen C.J., Starr C.E. (2008). Epidemiology of gram-negative conjunctivitis in neonatal intensive care unit patients. Am. J. Ophthalmol..

[7] Bremond-Gignac D., Chiambaretta F., Milazzo S.A. (2011). European perspective on topical ophthalmic antibiotics: Current and evolving options. Ophthalmol. Eye Dis..

[8] Horan T.C., Andrus M., Dudeck M.A. (2008). CDC/NHSN surveillance definition of health care-associated infection and criteria for specific types of infections in the acute care setting. Am. J. Infect. Control.

[9] Nixon H.K. (2018). Preparation of fortified antimicrobial eye drops. Kerala J. Ophthalmol..

[10] Dias C., Gonçalves M., João A. (2013). Epidemiological study of hospital-acquired bacterial conjunctivitis in a level III neonatal unit. Sci. World J..

[11] Borer A., Livshiz-Riven I., Golan A., Saidel-Odes L., Zmora E., Raz C., Melamed R., Plakht Y., Peled N. (2010). Hospital-acquired conjunctivitis in a neonatal intensive care unit: Bacterial etiology and susceptibility patterns. Am. J. Infect. Control.

[12] Al-Arosi S.A.H., Al-shamahi E.Y., Al-Kholani A.I.M., Al-Jawfi A.Y., Al-Shamahy H.A., Al-Moyed K.A.A., Al-Ankoshy A.A.M. (2021). Neonatal bacterial conjunctivitis in tertiary hospitals in Sana’a city, Yemen. Univ. J. Pharm. Res..

[13] Faulhaber F.R.S., Procianoy R.S., Silveira R.C. (2019). Side effects of phototherapy on neonates. Am. J. Perinatol..

[14] Tang X.J., He J.T., Liu Q., Chen X.K., Chen L. (2022). Severe ophthalmia neonatorum in Southwest China: A 5-year review of demographics, microbiological results, and risk factors. Int. Ophthalmol..

[15] Tang S., Li M., Chen H., Ping G., Zhang C., Wang S.A. (2017). A chronological study of the bacterial pathogen changes in acute neonatal bacterial conjunctivitis in southern China. BMC Ophthalmol..

